# Financial well-being and financial stress as predictors of overall well-being and life satisfaction among Peruvian workers

**DOI:** 10.3389/fpsyg.2025.1553006

**Published:** 2025-09-25

**Authors:** Oscar Mamani-Benito, María Celinda Cruz Ordinola, Roberto Dante Olazabal Boggio, Mariné Huayta-Meza, Edison Effer Apaza-Tarqui, Milagros Yesenia Pacheco Vizcarra, Wilter C. Morales-García

**Affiliations:** ^1^Facultad de Ciencias de la Salud, Universidad Señor de Sipán, Chiclayo, Peru; ^2^Facultad de Ciencias Empresariales, Universidad Peruana Unión, Juliaca, Peru; ^3^Instituto de Datos e Inteligencia Artificial, Universidad Ricardo Palma, Lima, Peru; ^4^Facultad de Ciencias Contables y Administrativas, Universidad Nacional del Altiplano, Puno, Peru; ^5^Dirección General de Investigación, Universidad Peruana Unión, Lima, Peru

**Keywords:** subjective well-being, life satisfaction, financial stress, financial well-being, Peru

## Abstract

**Background:**

The COVID-19 pandemic significantly impacted the mental health and overall well-being of the working population, exacerbating financial problems that influence subjective well-being and life satisfaction. Financial stress and financial well-being have emerged as key predictors of these dimensions.

**Objective:**

To determine whether financial well-being and financial stress are significant predictors of overall well-being and life satisfaction among Peruvian workers.

**Methods:**

A cross-sectional predictive study was conducted with 1,208 Peruvian workers aged 18–64 years (*M* = 25.26, SD = 7.99), using standardized instruments such as the General Well-Being Index, Financial Stress Scale, Satisfaction with Life Scale, and Financial Well-Being Scale.

**Results:**

The model demonstrated a good fit to the data (χ^2^ = 1399.76, *p* < 0.001, df = 269, CFI = 0.962, TLI = 0.958, RMSEA = 0.062, SRMR = 0.051). Financial well-being had a positive effect on overall well-being (β = 0.52, *p* < 0.001) and life satisfaction (β = 0.24, *p* < 0.001). Financial stress showed a negative effect on life satisfaction (β = −0.19, *p* < 0.001) but did not significantly affect overall well-being (β = −0.02, *p* > 0.001).

**Conclusion:**

Financial well-being plays a critical role in subjective well-being and life satisfaction, while financial stress negatively impacts cognitive evaluations of life. Occupational health strategies should incorporate financial education programs and measures to mitigate financial stress, fostering economic resilience and holistic well-being among workers.

## 1 Introduction

The economically active population has been facing various global challenges, one of the most recent being the COVID-19 pandemic, which has triggered a series of social and economic changes worldwide (Mack et al., 2021). This situation has significantly altered workers’ lifestyles, affecting their perceived quality of life (Zimpelmann et al., 2021) and mental health (Antipova, 2021).

Studies such as the one conducted by Salameh et al. (2020) have shown that financial difficulties increased stress and anxiety symptoms in a large portion of the economically active population. Other research also demonstrated that financial concerns were positively associated with mental distress (Wolfe and Patel, 2021), especially among vulnerable populations such as women, who were more likely to lose their jobs and experience alarming levels of anxiety and stress (Hossain, 2021).

In light of these findings, it is reasonable to assume that the working population has faced disruptions in their perception of well-being, particularly in their cognitive self-assessment of key aspects of their lives (Park et al., 2021).

### 1.1 Overall well-being and life satisfaction

Over time, the study of well-being has made significant contributions in areas such as physical health (Liyanage et al., 2023), organizational behavior (Lussier et al., 2025), teaching and learning (Özparlak et al., 2023), and mental health (Khalid and Syed, 2024). In terms of its interpretation, researchers have identified two classical traditions: the hedonic tradition, which focuses on the subjective experience of pleasure or “feeling good” (subjective well-being), and the eudaimonic tradition, which emphasizes personal growth and the realization of human potential (psychological well-being) (Ruggeri et al., 2020).

Regarding overall well-being, this variable represents the balance a person achieves across various aspects of life, such as physical and mental health, social relationships, and the environment in which they live (Calleja and Mason, 2020). Unlike subjective well-being, which involves a self-assessment of what each individual thinks and feels about their own life, overall well-being refers to a more holistic and multidimensional concept, including social, physical, and even environmental factors. These aspects go beyond mere subjective perception (Goodman, 2025), making overall well-being a broader and less restricted construct than other similar concepts.

Aligned with this perspective, the study of life satisfaction has also gained importance, as it constitutes a component of well-being—specifically, the cognitive component of subjective well-being (Jebb et al., 2020). Consequently, researchers define life satisfaction as a cognitive process that involves a self-assessment to evaluate the quality of life based on personal criteria and standards aligned with one’s current life circumstances (Calderón-De la Cruz et al., 2018), given its association with physical health (Potoczny et al., 2025), family satisfaction (Garnique-Hinostroza et al., 2024), interpersonal relationships (Zhao et al., 2024) and economic situation (Castellanos and Zapata-Antón, 2023).

Nevertheless, within the context of the health emergency, it has been observed that both well-being and life satisfaction levels among the economically active population have undergone significant changes, as revealed by studies conducted with Norwegian employees (Bakkeli, 2021). Another study in the same country found that restrictions on transportation, urban spaces, public areas, and medical or commercial services also negatively impacted perceptions of mental well-being (Mouratidis, 2021).

### 1.2 Predictors of overall well-being and life satisfaction

Various factors predict or explain the levels of well-being and life satisfaction in the working population. These range from variables such as age, gender, and relative wealth (Park et al., 2019) to health status, social relationships (Fu et al., 2022), personality traits, and work environment factors like job commitment and satisfaction (Marek et al., 2018). However, within the context of businesses and financial crises, such as the one caused by the COVID-19 pandemic (Doern, 2021), new evidence highlights significant manifestations experienced by dependent workers in Latin America, including financial well-being and financial stress (Robillard et al., 2020).

First, financial stress is defined as a subjective and unpleasant feeling experienced by individuals who perceive themselves as unable to meet economic demands, pay debts, or maintain sufficient funds for subsistence (Tito-Betancur et al., 2021). During the health emergency, this was often experienced as an emotional reaction to threats and uncertainty, resulting in physical and emotional tension (Carranza et al., 2021). In this regard, the Organización Internacional del Trabajo (2020) revealed that financial stress was one of the primary psychological manifestations during the COVID-19 pandemic, posing potential occupational risks and increasing the likelihood of workplace injuries and accidents (Ceballos-vásquez et al., 2020). Nevertheless, workers in other sectors such as education, transportation, commerce, and infrastructure have also shown manifestations of financial stress (Robillard et al., 2020), as revealed in a study indicating that income loss and financial strain influenced the risk of post-traumatic stress depending on occupation (Mejia et al., 2020).

Regarding its relationship with overall well-being and life satisfaction, previous studies suggest that a functional relationship between these variables is to be expected. For instance, Mejía (2017) concluded that financial well-being explained 60% of the variability in financial stress among workers in an oil company Berrill et al. (2020) found a negative association between financial difficulties, overall well-being, and life satisfaction in adults over 50 from 20 European countries. Additionally, Ryu and Fan (2022) reported that financial concerns were significantly associated with greater psychological distress among American adults. Moreover, Heo et al. (2020) found that financial stress reduced life satisfaction among U. S. farmers. Finally, Coşkuner Aktaş and Çiçek (2023) showed that life satisfaction was affected by the financial stress experienced by Turkish families during the COVID-19 pandemic.

Second, financial well-being refers to an individual’s perception of their ability to maintain a desired standard of living and achieve financial freedom (Foong et al., 2021). This concept has evolved to encompass both objective dimensions, such as material resources, income, and debt, and subjective dimensions, focusing on personal perceptions and satisfaction with one’s financial situation (Estela-Delgado et al., 2023). Current definitions and measurements of financial well-being are diverse and include multiple facets, integrating approaches that consider both the tangible aspects of personal finances and the subjective factors related to individual economic perceptions (Riitsalu et al., 2024).

Regarding its relationship with overall well-being, studies reveal that financial well-being is a key predictor of mental well-being (Ahamed, 2024). This was confirmed by other investigations that identified a functional relationship between both variables. For example, a study conducted with older adults in Malaysia found a strong correlation (Foong et al., 2021), and another study with cancer patients in Hong Kong revealed that low financial well-being relatively moderated the direct impact of health-related living conditions on physical and functional well-being (So et al., 2023). As for its relationship with life satisfaction, a study carried out with entrepreneurs in South Africa found that investors who perceived themselves as having high financial well-being were more likely to report high life satisfaction (Dickason et al., 2019). This finding was later confirmed by other studies conducted in China, which discovered that economic and social parameters affect the life satisfaction of the general population (Su and Muhammad, 2023), as well as by another study conducted with agricultural households, confirming that higher relative income was associated with a higher level of life satisfaction (Hong et al., 2023).

### 1.3 Justification

After enduring a grueling health crisis for over 2 years, it is expected that the active working population will regain a sense of well-being and develop a positive perception of life satisfaction. These variables are key indicators of mental health and have a decisive influence on a country’s economic growth. In this context, and given the lack of predictive models to understand the new predictors of overall well-being and life satisfaction in a post-pandemic scenario, this research addresses this gap in knowledge. It proposes a predictive model that serves as a foundation for implementing strategies to prevent occupational risks and promote occupational health.

### 1.4 Hypotheses

Based on the discussion, the following research hypotheses are proposed:

- H1: Financial well-being has a positive effect on overall well-being.
- H2: Financial stress has a negative effect on overall well-being.
- H3: Financial well-being has a positive effect on life satisfaction.
- H4: Financial stress has a negative effect on life satisfaction.

### 1.5 Objective

To determine whether financial well-being and financial stress are significant predictors of overall well-being and life satisfaction among Peruvian workers.

## 2 Method

### 2.1 Design

A predictive and cross-sectional study was conducted (Ato et al., 2013).

### 2.2 Participants

The study population consisted of economically active adults. In this case, a segmentation was applied based on four age groups: youth, early adulthood, middle adulthood, and old age, which are traditional criteria according to developmental psychology (Stassen, 2025). Youth includes individuals in training or at the beginning of their working life; early adulthood is marked by greater stability and professional development; middle adulthood is often associated with increased financial responsibilities; and old age involves the transition toward retirement (Novella et al., 2018), which in Peru occurs at age 65 for both the public and private systems.

Using a non-probabilistic purposive sampling method, 1,208 workers (50.7% men) voluntarily participated from the three regions of Peru (Highlands = 84.5%, Jungle = 5.0%, and Coast = 10.4%). All participants were adults, including adolescents and young people between 18 and 29 years old (83.7%), early-stage adults aged 30 to 39 (10.1%), mid-stage adults aged 40 to 59 (6%), and adults nearing retirement over 60 years old (0.2%). The 81.8% of participants have a higher education level, 25.6% completed secondary education, 1.9% had no formal education, and 0.7% had only primary education. Additionally, 57.7% were salaried employees, while 42.3% were self-employed, managing or operating small or medium-sized enterprises. Inclusion criteria required participants to be adults, have dependent or independent employment, and complete the entire survey. Exclusion criteria included failure to complete the questionnaire and refusal to provide informed consent.

### 2.3 Instrument

Satisfaction with Life Scale (SWLS, Caycho-Rodríguez et al., 2018). This scale consists of 5 items organized into a single factor, with Likert response options ranging from 1 (Strongly Disagree) to 5 (Strongly Agree). In this study, the SWLS showed acceptable reliability, α = 0.89.

Financial Stress Scale (EFT-Cov19, Carranza et al., 2021). Comprising 7 items distributed in a single factor, this scale uses a 5-point Likert response format: 1 = Strongly Disagree, 2 = Disagree, 3 = Neutral, 4 = Agree, 5 = Strongly Agree. In this study, the scale demonstrated good internal consistency, α = 0.90.

General Well-Being Index (WHO-5, Caycho-Rodríguez et al., 2020). This index consists of 5 items organized into a single factor, with Likert response options ranging from 0 (Never) to 3 (Always). In this study, the reliability was α = 0.87.

Financial Well-Being Scale (Condori and Vidalon, 2020). This scale contains 8 items distributed in a single factor, with 5-point Likert response options ranging from 1 (Strongly Disagree) to 5 (Strongly Agree). In this study, the reliability was α = 0.71.

### 2.4 Procedures

Data were collected through an online questionnaire available from October 3 to December 12, 2023. The first section of the survey presented the study’s objectives and an informed consent form requesting voluntary participation. Participants were informed that their responses would remain strictly anonymous.

### 2.5 Data analysis

The data analysis was conducted using the “R” software, version 4.0.5, with the “lavaan” package (Rosseel, 2012). The factorial structure of the instruments was first examined using confirmatory factor analysis (CFA). Given the ordinal nature of the items, polychoric correlation matrices and the WLSMV estimator were used, which is recommended for ordinal measurement scales (Lei and Wu, 2012). The study’s predictive model was analyzed using the MLM estimator, which is suitable for numerical variables and robust against deviations from inferential normality (Muthen and Muthen, 2017). Model fit was evaluated using the Comparative Fit Index (CFI), Root Mean Square Error of Approximation (RMSEA), and Standardized Root Mean Square Residual (SRMR). The criteria for acceptable fit were CFI > 0.90 (Bentler, 1990), RMSEA < 0.080 (MacCallum et al., 1996), and SRMR < 0.080 (Browne and Cudeck, 1992). The predictive model was analyzed while controlling for the demographic variables of gender and age. Internal consistency reliability was assessed using Cronbach’s Alpha (α).

### 2.6 Ethical considerations

The study was approved by the ethics committee of Universidad Señor de Sipán (Code 710-CIEI).

## 3 Results

Table 1 presents indicators for the mean, standard deviation, skewness, kurtosis, and the correlation matrix. Correlation values ranged between 0.17 and 0.56, representing significant low-to-moderate associations. The table also shows the internal consistency of each scale, as measured by Cronbach’s Alpha, with values ranging from 0.71 to 0.90, indicating very good reliability.

**Table 1:** Descriptive statistics, internal consistencies, and correlations of study variables.

Variables	*M*	DE	*A*	*K*	α	1	2	3	4
1. Life satisfaction	14.43	4.66	0.21	−0.30	0.89	1			
2. General well-being	16.53	3.13	−0.24	−0.21	0.87	0.56**	1		
3. Financial well-being	23.67	4.09	0.01	0.55	0.71	−0.38**	−0.43**	1	
4. Financial stress	18.93	6.51	0.03	−0.38	0.90	0.00	0.17**	0.28**	1

**Figure 1:**
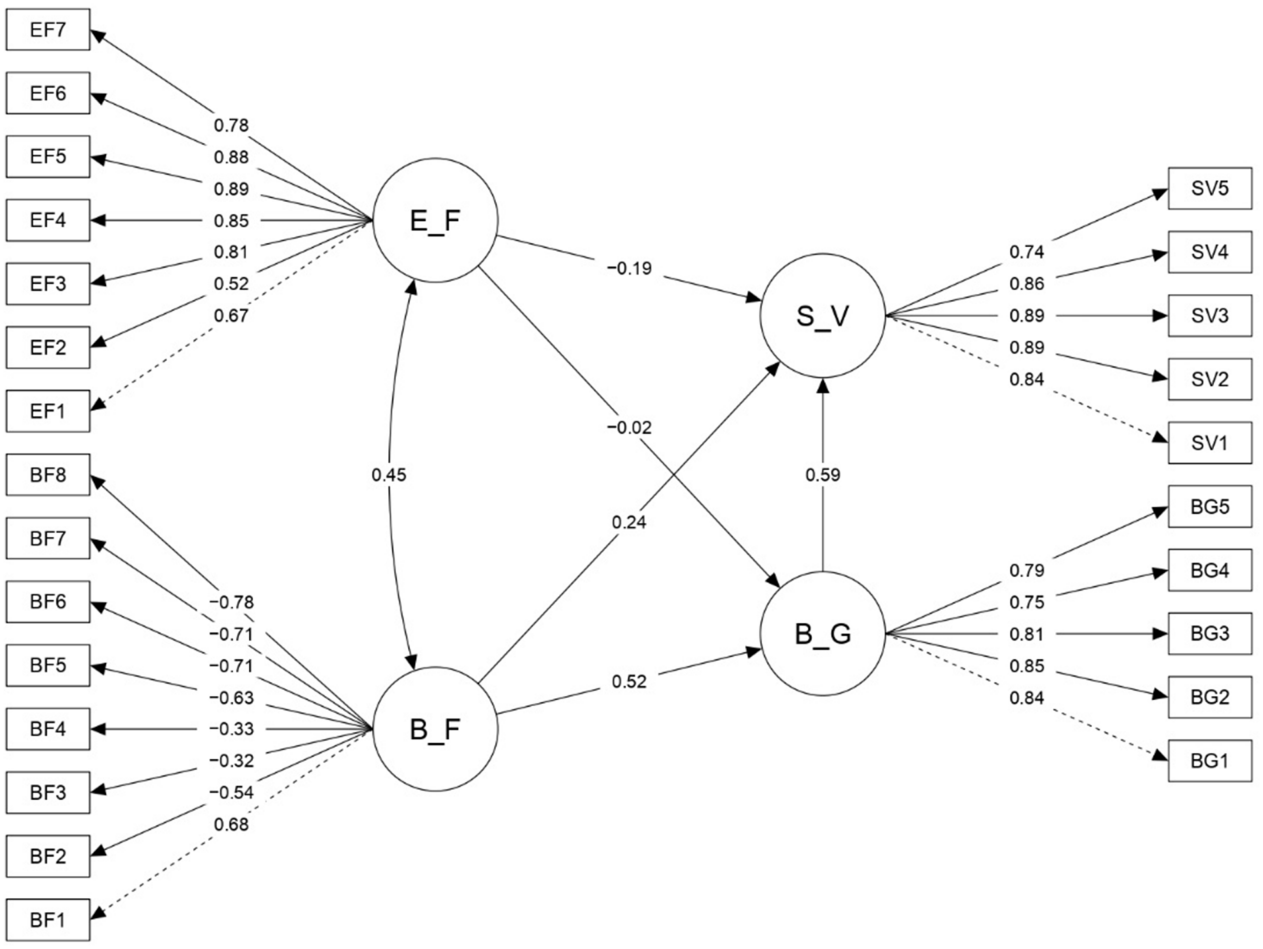
Predictive model. E_F = Financial stress, B_F = Financial well-being, S_V = Life satisfaction, B_G = Overall well-being.

The SEM analysis showed that the predictive model fit the data well: χ^2^ = 1399.76, *p* < 0.001, df = 269, CFI = 0.962, TLI = 0.958, RMSEA = 0.062, SRMR = 0.051. These results confirmed H1, H3, and H4, indicating that financial well-being positively affects overall well-being (β = 0.52, *p* < 0.001) and life satisfaction (β = 0.24, *p* < 0.001), while financial stress negatively affects life satisfaction (β = −0.19, *p* < 0.001). However, the negative effect of financial stress on overall well-being (β = −0.02, *p* > 0.001) was not supported. The standardized solution is illustrated in Figure 1.

## 4 Discussion

The COVID-19 pandemic has significantly impacted the mental health of the working population, with factors such as the lack of financial well-being and financial concerns playing a key role in this negative effect. In this context, the present study aimed to determine whether financial well-being and financial stress are significant predictors of overall well-being and life satisfaction among Peruvian workers.

Regarding H1, it was hypothesized that financial well-being has a positive effect on overall well-being. The results support this hypothesis, as a large positive effect size was observed. This finding aligns with previous studies, which suggest that individuals who maintain control over their finances and can meet current and future needs tend to evaluate their lives positively overall (Netemeyer et al., 2018). Recent research supports this interpretation, emphasizing that financial events such as those stemming from the pandemic can influence mental well-being. While some individuals adapted to new life circumstances, others did not, highlighting the role of subjective evaluations in determining the impact of life events (Chilver et al., 2023). These findings suggest that effective management of income and expenses significantly influences mental well-being. Financial capability not only positively affects psychological health but may do so regardless of the absolute level of income (Ryu and Fan, 2022).

Regarding H2, it was hypothesized that financial stress has a negative effect on overall well-being. Based on the results, this hypothesis cannot be accepted, as the effect size was nearly zero. As such, this finding raises some uncertainty and may even suggest that the present study underestimates the effect of financial stress on overall well-being. This is particularly notable considering that previous studies provided evidence supporting the idea that financial difficulties and concerns affect individuals’ perceptions of balance and satisfaction across various life domains (Berrill et al., 2020; Ryu and Fan, 2022). Nevertheless, this result may be understood in light of three considerations. First, financial stress is a reactive and temporary emotional state, which may have a greater impact on mental health indicators rather than on global well-being, which is a broader and more stable construct (Simonse et al., 2022). Second, the effects of financial stress are often mediated through intermediary variables, and thus its influence may be expressed through psychological and contextual mediators (Nassir et al., 2025). Third, 83.7% of the participants in this study were young and early adults, who generally have a greater perception of time and future opportunities (Rubin et al., 2024), and therefore may perceive economic problems as temporary and less threatening to their overall well-being.

For H3, it was hypothesized that financial well-being positively affects life satisfaction. The results confirm this hypothesis, revealing a moderate positive effect size. Consistent with prior research, financial security and freedom, which involve maintaining control over daily and monthly finances, positively impact individuals’ evaluations of their lives, including aspects such as family, education, work, health, and social relationships (Copur and Dogan, 2023). This finding adds to existing evidence from studies on economically active women (Foong et al., 2021), financial investors (Dickason et al., 2019), and salaried workers (Ortiz et al., 2019). Furthermore, the results align with theories suggesting that sound financial management practices and financial satisfaction are important precursors to life satisfaction (Atatsi et al., 2023). As Kumar et al. (2024) argue, consistent and well-managed household income enhances life satisfaction for all family members.

Finally, regarding H4, it was hypothesized that financial stress negatively affects life satisfaction. This hypothesis was supported, although the effect size was minimal yet statistically significant. Previous studies have shown that financial stress can deeply impact subjective evaluations of one’s current and future financial life (Fan and Henager, 2022). Struggles to pay bills, buy necessities such as food and clothing, secure adequate housing, cover utilities, access healthcare, and manage transportation costs clearly influence the cognitive component of subjective well-being (Bialowolski et al., 2021). This finding is consistent with research indicating that financial stress has a greater impact on life satisfaction among older adults (Hou et al., 2022), as well as active workers (Heo et al., 2020), adolescents (Meyer et al., 2021), and university students (Kim, 2024). Insufficient monetary resources and overwhelming debt can impose additional psychological burdens, further deteriorating overall health and psychological well-being (Sommet and Spini, 2022). However, recent studies suggest that economic crises tend to have a lesser impact on life satisfaction in populations of high-trust countries compared to low-trust ones (Clench-Aas et al., 2021).

### 4.1 Implications

In Peru, the employment pattern shows a high participation of adults and young people in early and intermediate life stages (Instituto Nacional de Estadistica e Informatica, 2025). This is reflected in the composition of the present study’s sample, where 83.7% are young individuals (18–29 years old) and 10.1% are young adults (30–39 years old), in contrast to 6% who are middle-aged adults and only 0.2% who are nearing retirement. From this age predominance, it can be inferred that the economically active population in this study has developed a more optimistic perception of time and future opportunities (Rubin et al., 2024), which may support the interpretation that economic difficulties are seen as temporary. In contrast, the low proportion of adults nearing retirement suggests that our findings should be interpreted with caution for this age group, which is likely experiencing greater financial challenges related to the proximity of retirement (Diario Gestión, 2025).

Based on this and other findings from the present study, it is necessary to recognize that political administrators, mental health professionals, and financial experts can benefit from the idea that mental health and financial security go hand in hand when integrating financial security into occupational health programs. The following implications are highlighted:

First, our results confirm the importance of ensuring financial security during times of not only health crises but also social crises. In this context, workers leading financially vulnerable households are those most likely to experience the economic consequences. Second, the government and relevant institutions should include economically vulnerable groups in mental health programs that is, include financially vulnerable households and address their specific mental health needs. Third, mental health interventions and strategies should address not only the psychological symptoms of COVID-19 but also include financial stress and financial well-being, through financial counseling and training, especially for financially vulnerable households. Finally, as an important lesson and reflection for future health and economic crises, it is essential to promote saving and avoid unnecessary debt. This can make households more resilient to the adverse mental health consequences of future crises.

### 4.2 Limitations

The study presented some limitations. First, the use of a non-random sample with a predominance of participants from the highlands limits the ability to generalize the results to the population of all three regions of Peru, since the characteristics and circumstances of the sample may differ from those of the broader Peruvian workforce. In light of this, it is recommended that future studies employ probabilistic sampling methods in order to increase the sample size and diversify recruitment sources, ensuring a more balanced representation by region. Second, the data were collected through an online form. Although this method allows for a larger number of participants, it also introduces potential selection biases, as only individuals with internet access were able to participate. This excludes workers who, due to geographic or digital literacy barriers, do not have regular access to online platforms. To address this, future research is encouraged to include face-to-face surveys, especially in regions with low internet connectivity. Third, regarding the measurement of financial stress, although we used a scale with favorable psychometric properties, the initial validation process was carried out primarily with participants from the coastal region. In contrast, the majority of participants in the present study are from the highlands. This discrepancy suggests that the scores obtained may not fully reflect the subjective and cultural experience of financial stress among adults from the highlands, due to potential sociocultural, economic, and linguistic differences not considered in the original validation sample.

Therefore, further studies are necessary to evaluate whether the results hold true for participants from other regions of Peru. Finally, since the data were collected in a cross-sectional manner, it is not possible to establish a temporal sequence between the study variables, which is essential for inferring causal relationships. In this case, our results indicate functional relationships suggesting a significant influence between the independent and dependent variables; however, this does not allow for the establishment of causality for the observed effects. Although this study aimed to clarify the relationship between financial stress and well-being through a moderation analysis using demographic variables, the results were not favorable. In light of this, it is imperative that future studies implement longitudinal or experimental designs to validate causality and support evidence-based intervention strategies.

## 5 Conclusion

In conclusion, when workers maintain control over their finances and meet their current and future needs, they are more likely to perceive their lives positively in general terms. Conversely, living under economic distress and uncertainty due to financial difficulties can negatively affect their self-assessment in specific areas such as family, education, work, health, friendships, and leisure time.

## Data Availability

The raw data supporting the conclusions of this article will be made available by the authors, without undue reservation.
